# AZD8055 Is More Effective Than Rapamycin in Inhibiting Proliferation and Promoting Mitochondrial Clearance in Erythroid Differentiation

**DOI:** 10.1155/2024/2639464

**Published:** 2024-10-08

**Authors:** Qian Liu, Tao Hao, Ze Lin, Yipeng Fang, Lei Li, Daqi Huang, Jianbo Wu, Yanchao Zhao, Xin Zhang

**Affiliations:** ^1^Department of Cardiology, The Binzhou Medical University Hospital, Binzhou, Shandong Province, China; ^2^Department of Colorectal and Anal Surgery, The Binzhou Medical University Hospital, Binzhou, Shandong Province, China; ^3^Shantou University Medical College, Shantou, Guangdong Province, China; ^4^Department of Cardiology, The First Affiliated Hospital of Shantou University Medical College, Shantou, Guangdong Province, China; ^5^Department of Critical Care Medicine, Tianjin Medical University General Hospital, Tianjin, China; ^6^Binzhou Health Commission, Binzhou, Shandong Province, China; ^7^Laboratory of Medical Molecular Imaging, The First Affiliated Hospital of Shantou University Medical College, Shantou, Guangdong Province, China

## Abstract

**Background:** As an important downstream effector of various signaling pathways, mTOR plays critical roles in regulating many physiological processes including erythropoiesis. It is composed of two distinct complexes, mTORC1 and mTORC2, which differ in their components and downstream signaling effects. Our previous study revealed that the inhibition of mTORC1 by rapamycin significantly repressed the erythroid progenitor expansion in the early stage but promoted enucleation and mitochondria clearance in the late stage of erythroid differentiation. However, the particular roles and differences of mTORC1 and mTORC2 in the regulation of erythropoiesis still remain largely unknown. In the present study, we investigated the comparative effects of dual mTORC1/mTORC2 mTOR kinase inhibitor AZD8055 and mTORC1 inhibitor rapamycin on erythroid differentiation in K562 cells induced by hemin and erythropoiesis in β-thalassemia mouse model.

**Materials and Methods:** In vitro erythroid differentiation model of hemin-induced K562 cells and β-thalassemia mouse model were treated with AZD8055 and rapamycin. Cell Counting Kit-8 was used to detect cell viability. The cell proliferation, cell cycle, erythroid surface marker expression, mitochondrial content, and membrane potential were determined and analyzed by flow cytometry and laser scanning confocal microscopy. Globin gene expression during erythroid differentiation was measured by RT-qPCR. The mTORC2/mTORC1 and autophagy pathway was evaluated using western blotting.

**Results:** Both AZD8055 and rapamycin treatments increased the expression levels of the erythroid differentiation-specific markers, CD235a, *α*-globin, *γ*-globin, and *ε*-globin. Notably, AZD8055 suppressed the cell proliferation and promoted the mitochondrial clearance of hemin-induced K562 cells more effectively than rapamycin. In a mouse model of *β*-thalassemia, both rapamycin and AZD8055 remarkably improve erythroid cell maturation and anemia. Moreover, AZD8055 and rapamycin treatment inhibited the mTORC1 pathway and enhanced autophagy, whereas AZD8055 enhanced autophagy more effectively than rapamycin. Indeed, AZD8055 treatment inhibited both mTORC2 and mTORC1 pathway in hemin-induced K562 cells.

**Conclusion:** AZD8055 is more effective than rapamycin in inhibiting proliferation and promoting mitochondrial clearance in erythroid differentiation, which might provide us one more therapeutic option other than rapamycin for ineffective erythropoiesis treatment in the future. These findings also provide some preliminary information indicating the roles of mTORC1 and mTORC2 in erythropoiesis, and further studies are necessary to dissect the underlying mechanisms.

## 1. Introduction

Erythropoiesis is a multistep and hematopoietic process tightly regulated by cell lineage specification, proliferation, and differentiation. The study of erythropoiesis was crucial for understanding and developing treatments for many erythroid disorders including β-thalassemia and leukemia. Leukemia is a kind of hematopoietic stem cell malignant clonal disease, characterized by uncontrolled proliferation and inhibition of erythropoiesis. Chronic myelocytic leukemia (CML) accounts for about 20% of all adult leukemia. K562, a cell line established from the pleural fluid of female CML patients, has stem cell characteristics. K562 cells are important cell line models for researching red blood cells, granulocytes, and monocyte-related diseases and treatment mechanisms in vitro and can induce differentiation under specific conditions [1]. As a heme oxidation product, hemin induces erythroid differentiation and expression of corresponding markers in K562 cells, thereby increasing the expression of glycoproteins, erythroid transcription factor, GATA-1, and hemoglobin expression [2, 3]. By detecting these markers, we can understand the direction and stage of the erythroid differentiation of K562 cells and provide a scientific basis for the maturation mechanism.

Autophagy is critical for removing unnecessary organelles such as mitochondria and ribosomes, enabling erythroid progenitors to differentiate into mature erythrocytes [4]. It is mainly regulated by autophagy-related proteins and pathways, with the mTORC1-dependent pathway being the key negative regulatory [5]. This pathway is essential for cell metabolism and growth. As an important downstream effector of various signaling pathways, mTOR plays critical roles in regulating many physiological processes including erythropoiesis. Our previous research showed that rapamycin, an mTORC1 inhibitor, inhibited proliferation in early human erythroid cells and promoted the clearance of mitochondria during late differentiation [6]. However, rapamycin may not completely inhibit the phosphorylation of mTORC1 [7]. In addition, inhibition of mTORC1 activates mTORC2, weakening the effect of mTORC1 inhibitors. mTORC1 and mTORC2 regulate each other [6]; therefore, mTORC2, as an important upstream regulator of AKT phosphorylation activity, regulates the P13K/AKT/mTORC1 signaling pathway, whereas S6K, the downstream target of mTORC1, negatively regulates mTORC2, resulting in a negative feedback [8]. Targeted mTORC2 inhibition does not affect the mTORC1-dependent negative feedback loop and offers a more acceptable therapeutic window.

Moreover, mTORC2 has been identified as a promising therapeutic target in cancer biology studies [9]. However, the mTORC2 assembly and 3D structure remain unknown; therefore, specific mTORC2 inhibitors have not been developed yet. As a new generation of mTOR inhibitors has demonstrated comprehensive and durable inhibition of mTORC1 and mTORC2, the results have been more effective than those obtained with rapamycin. Consequently, they are currently used for cancer treatment in preclinical and clinical trials [10, 11]. The mTORC2/mTORC1 inhibitor, AZD8055, an orally available, potent, and specific mTOR inhibitor that affects both mTORC1 and mTORC2 complexes, is a more active kinase inhibitor than rapamycin. Although AZD8055 can effectively kill cancer cells in tumors, erythroid differentiation and its effect on humans have not been thoroughly investigated. Therefore, in this study, we to compare the roles of AZD8055 and rapamycin on erythroid differentiation of hemin-induced K562 cells and β-thalassemia mouse model, which may provide some preliminary information indicating the roles of mTORC1 and mTORC2 in erythropoiesis, and further studies are necessary to dissect the underlying mechanisms.

## 2. Materials and Methods

### 2.1. Cell Culture

The human CML cell line K562 was purchased from Pricella Life Technology (Wuhan, China). Cells were cultured in IMDM medium supplemented with 10% fetal bovine serum (Hyclone, Logan, UT, USA) and 100 μg/mL penicillin-streptomycin (HyClone). The K562 cells were cultured in a 5% CO_2_ incubator at 37°C. K562 cells were treated with 40 μmol/L hemin (Sigma, St. Louis, MO, USA) for erythroid differentiation. Rapamycin and AZD8055 were purchased from MedChemExpress (Shanghai, China). The cells were treated with the indicated concentrations, whereas the cells in the control group received an equal volume of DMSO (Sigma).

### 2.2. Mice

Hbb-bs&Hbb-bt DKO mice model for β-thalassemia (Cyagen, Sunzhou, China)and 12-week-old C57Bl6 mice were used in all experiments. Protocols were approved by the Institutional Animal Care and Use Committee of the First Affiliated Hospital of Shantou University Medical College. Mice received intraperitoneal administration at a dose of 4 mg/kg body weight of rapamycin or 10 mg/kg AZD8055 daily for 2 weeks. Blood samples were obtained from the inner canthus vein and collected in EDTA or heparin. Complete blood counts (CBC) were measured with an ADVIA 120 Hematology analyzer.

### 2.3. CCK8

Cell Counting Kit-8 (CCK-8, MedChemExpress) was used to evaluate cell proliferation. In brief, K562 cells were plated onto 96-well cell culture plates at a density of 5000 cells/well and treated with different concentrations of chemicals. After culturing for 24 or 48 h, 10 µL of CCK-8 reagent was added to each well. Finally, the cells were incubated for 1 h at 37°C before measuring the optical density at 450 nm. The experiments were performed in triplicate.

### 2.4. Flow Cytometry

The cell cycle was analyzed using a Cell Cycle and Apoptosis Analysis Kit (FXP021, 4A Biotech Co. Ltd, Beijing, China). Cell proliferation was monitored using a carboxyfluorescein diacetate succinimidyl ester (CFDA-SE) Cell Proliferation Assay Kit (Beyotime, Shanghai, China). Reactive oxygen species (ROS) were analyzed using 5-(and-6)-chloromethyl-2′,7′-dichlorodihydrofluorescein diacetate, acetyl ester (H2DCF-DA, BestBio, Shanghai, China). Mitochondrial membrane potential (MMP) was measured using the MitoProbe JC-1 Assay Kit (Invitrogen, Carlsbad, CA, USA). In addition, cell cycle and proliferation were measured using the BrdU-APC/7-AAD Kit (BD Biosciences, Franklin Lakes, NJ, USA). Flow cytometry data were analyzed using the FlowJo 8.0 software (GraphPad Prism, Inc., La Jolla, CA, USA). Cell cycle data were analyzed using the ModFit LT 3.0 software. Bone marrow *r* single cell suspensions were prepared and maintained in IMDM115% FBS, washed twice, preincubated with 10% rat serum, and stained with CD44-APC and TER119-FITC antibodies (BD Biosciences).

### 2.5. Confocal Microscopy

Phycoerythrin-conjugated antihuman CD235a (clone: HIR2, Thermo Fisher Scientific, Waltham, MA, USA) was used for cell-surface marker detection. MitoTracker and Hoechst 33342 (Beyotime) were used to localize the lysosomes and nucleus. The MitoProbe JC-1 Assay Kit (Invitrogen) was used to identify the MMP. Images were acquired using an Olympus FV1200MPE confocal microscope.

### 2.6. Quantitative Reverse-Transcription PCR (RT-qPCR)

Total RNA was extracted using an EasyPure RNA Kit, according to the manufacturer's instructions. cDNA was synthesized using the PrimeScript First Strand cDNA Synthesis Kit (Takara Bio, Dalian, China). PerfectStartTM Green qPCR SuperMix (TransGen Biotech, Beijing, China) was adopted for the qRT-PCR system as previously described [6]. The relative mRNA levels were normalized to those of β-actin. The primer sequences for the real-time PCR are listed in Table S1.

### 2.7. Western Blot Assay

Extraction of total proteins, separation on 10% sodium dodecyl sulfate–polyacrylamide gel electrophoresis, and detection were performed as previously described [6]. The primary antibodies against mTOR (#2983), p-mTOR (#5536), AKT (#4691), p-AKT (#4060), p70S6K (#9208), p-p70S6K-Thr389 (#9234), p-S6-Ser235/236 (#4858), S6 (#2217), LC3 (#12741), p62 (#8025), Beclin (#3495), and β-actin (#8457) were purchased from Cell Signaling Technology (Boston, USA).

### 2.8. Statistical Analyses

The results are presented as mean ± standard error (SD) of three independent experiments. Treatment effects were analyzed using a Student's *t*-test when comparing two variables, whereas intergroup and intragroup comparisons were conducted using one-way ANOVA with the Dunnett test. Results were considered statistically significant at *P* < 0.05.

## 3. Results

### 3.1. Viability of Hemin-Induced K562 Cells Was Suppressed More Effectively With AZD8055 Than With Rapamycin

**Figure 1:**
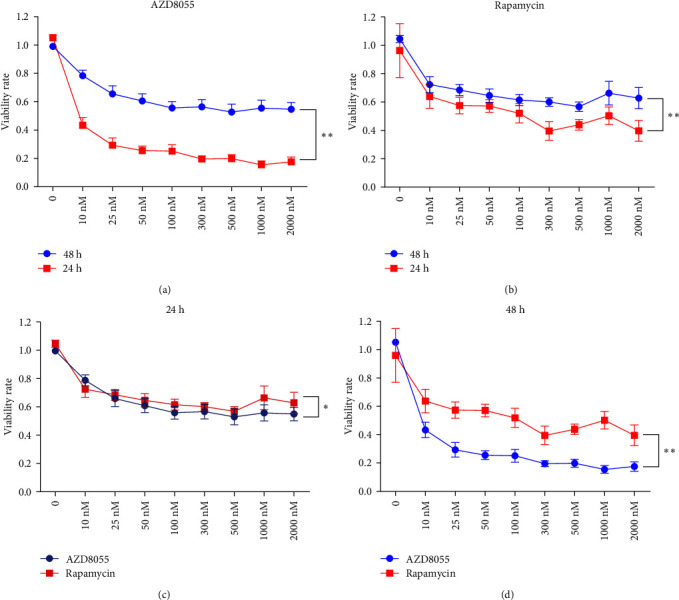
AZD8055 and rapamycin inhibited cell viability in a time- and concentration-dependent manner. (A) Hemin-treated K562 cells were treated with the indicated concentrations of AZD8055 for 24 and 48 h. (B) Hemin-treated K562 cells were treated with the indicated concentrations of rapamycin 24 and 48 h. (C) Hemin-treated K562 cells were treated with the indicated concentrations of AZD8055 or rapamycin for 24 h. (D) K562 cells were treated with the indicated concentrations of AZD8055 or rapamycin 24 h.  ^*∗*^*P* < 0.05,  ^*∗∗*^*P* < 0.01.

We assessed the effects of rapamycin and AZD8055 on hemin-induced K562 viability using the CCK-8. AZD8055 and rapamycin inhibited cell viability in a time- and concentration-dependent manner (Figure 1A, B). The inhibitory effect of AZD8055 was more marked than rapamycin at the same concentration and time (Figure 1C, D).

### 3.2. AZD8055 Had a Stronger Inhibitory Effect on Cell Proliferation Than Rapamycin

**Figure 2:**
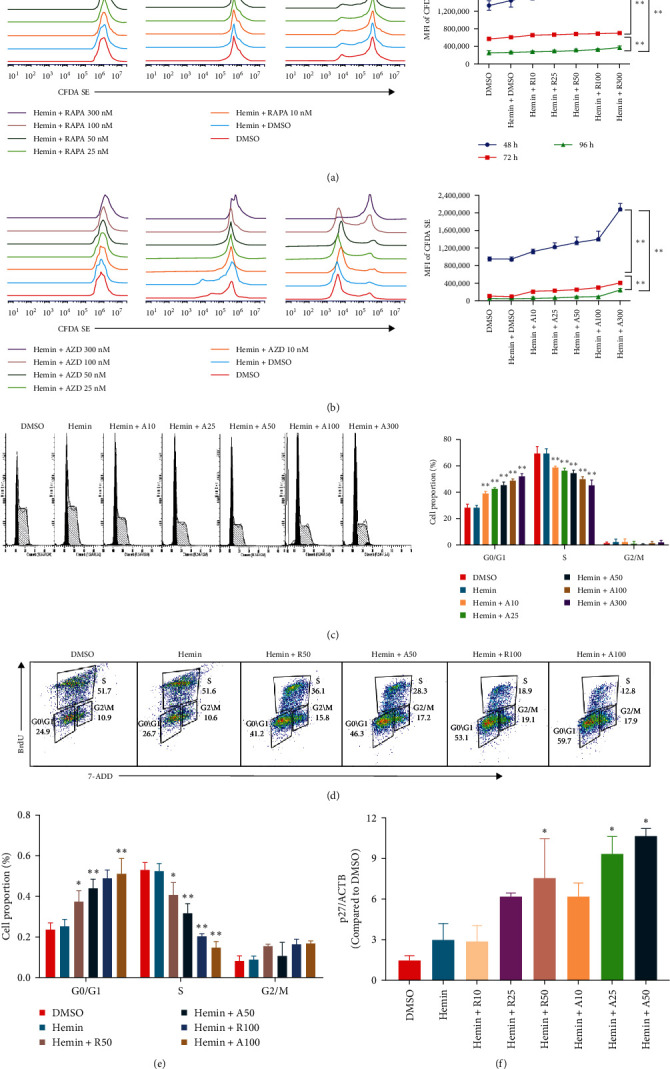
Stronger inhibitory effect of AZD8055 on cell proliferation than that of rapamycin. (A and B) Proliferation of cells was evaluated using flow cytometry after labeling with carboxyfluorescein diacetate succinimidyl ester (CFDA-SE), following treatment with rapamycin (A) or AZD8055 (B). The right panel shows the mean fluorescence intensity (MFI) of CFDA-SE. CFDA-SE fluorescence intensity decreases gradually with cell division and proliferation. (C) The K562 cells treated with rapamycin or AZD8055 were stained with propidium iodide and subjected to flow cytometry to analyze DNA content. The right panel shows the fractions (%) of cells in G0/G1, S, and G2/M phases. (D) K562 cells treated with rapamycin or AZD8055 for 72 h were stained with BrdU/7ADD and subjected to flow cytometry to analyze the cell cycle. (E) The right panel shows fractions (%) of cells in G0/G1, S, and G2/M phases. (F) p27 mRNA expression in K562 cells treated with rapamycin or AZD8055, as analyzed using RT-qPCR. Gene expression was normalized to beta-actin (ACTB). Data are the mean ± SD of technical triplicates from one of several independent experiments.  ^*∗*^*P* < 0.05,  ^*∗∗*^*P* < 0.01 vs. K562 cells treated with hemin. AZD (A), AZD8055; RAPA (R), rapamycin.

We performed CFDA-SE staining to detect cell proliferation using flow cytometry because cells undergoing division maintain half of the CFDA-SE fluorescence staining intensity of the parent cells. The mean fluorescence intensity (MFI) of rapamycin and AZD8055-treated cells increased with the increase in concentration and decreased with the increase in treatment time (Figure 2A, B), which indicated that AZD8055 and rapamycin could inhibit cell proliferation in a time- and concentration-dependent manner.

The effect of AZD8055 on the cell cycle was detected via flow cytometry using propidium iodide staining (Figure 2C). The results showed a considerable increase in cells blocked in the G0/G1 phase and a decrease in the S phases with the increase in AZD8055 concentration. To further confirm the above results, the effects of rapamycin and AZD8055 on the hemin-induced K562 cell cycle were analyzed using the BrdU and 7-AAD double staining method (Figure 2D and E). The results showed that with the increase in AZD8055 and rapamycin concentration, the proportion of cells in the S phase decreased gradually, whereas those in the G0/G1 phase gradually increased. At the same concentration, the proportion of cells in the S phase was lower after the AZD8055 treatment than after rapamycin treatment. Hemin, which induced K562 erythroid differentiation, did not affect the proportional effect of the cell cycle. Simultaneously, the cell cycle-related gene *p27* was upregulated in a concentration-dependent manner (Figure 2F). These findings demonstrated that cell cycle arrest was the primary mechanism by which AZD8055 and rapamycin suppressed cell proliferation.

### 3.3. Rapamycin and AZD8055 Did Not Exhibit a Significantly Different Effect on the Differentiation Ability of Erythrocytes

**Figure 3:**
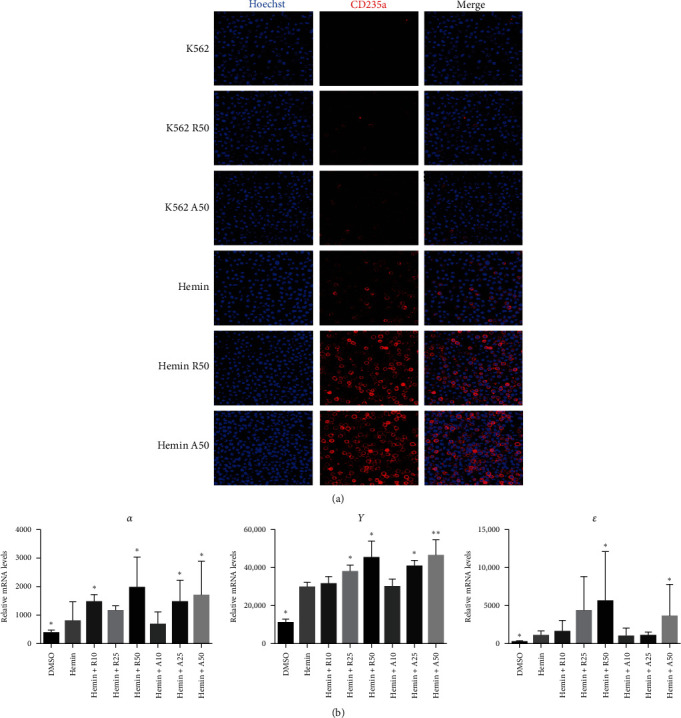
Lack of significant differences between rapamycin and AZD8055 on the differentiation ability of erythrocytes. (A) Confocal laser scanning microscopy images showing nucleus (blue) staining using Hoechst 33342 and CD235a (red) staining using PE-conjugated CD235a antibody in K562 cells treated with rapamycin or AZD8055 with or without hemin. (B) α-, γ-, and ε-globin mRNA expression in K562 cells treated with rapamycin or AZD8055. Data are the mean ± SD of technical triplicates from one of several independent experiments.  ^*∗*^*P* < 0.05,  ^*∗∗*^*P* < 0.01 vs. K562 cells treated with hemin. R, rapamycin; A, AZD8055.

CD235a is a marker of erythroid differentiation ability. After hemin treatment, the expression of CD235a was significantly increased. Both rapamycin and AZD8055 increased the expression of CD235a in K562 cells and hemin-induced K562 cells (Figure 3A). However, there was no significant difference in the expression of CD235a between the two drugs at the same concentration.

The typical markers of erythroid differentiation are α-, γ-, and ε-globin. The amount of γ-globin is an index to measure the differentiation ability of erythrocytes. RT-PCR showed that the expression levels of α-, γ-, and ε-globin were higher than those of the control group (Figure 3B). All the embryo–fetal globin genes were expressed in the hemin-induced K562 cells with increasing concentrations of rapamycin and AZD8055. The effects of AZD8055 on the accumulation of globin mRNA prompted us to verify its effect on human erythroid precursor cells, even when it has not shown higher effectiveness than rapamycin.

### 3.4. AZD8055 Promoted More Effective Mitochondrial Clearance Than Rapamycin

**Figure 4:**
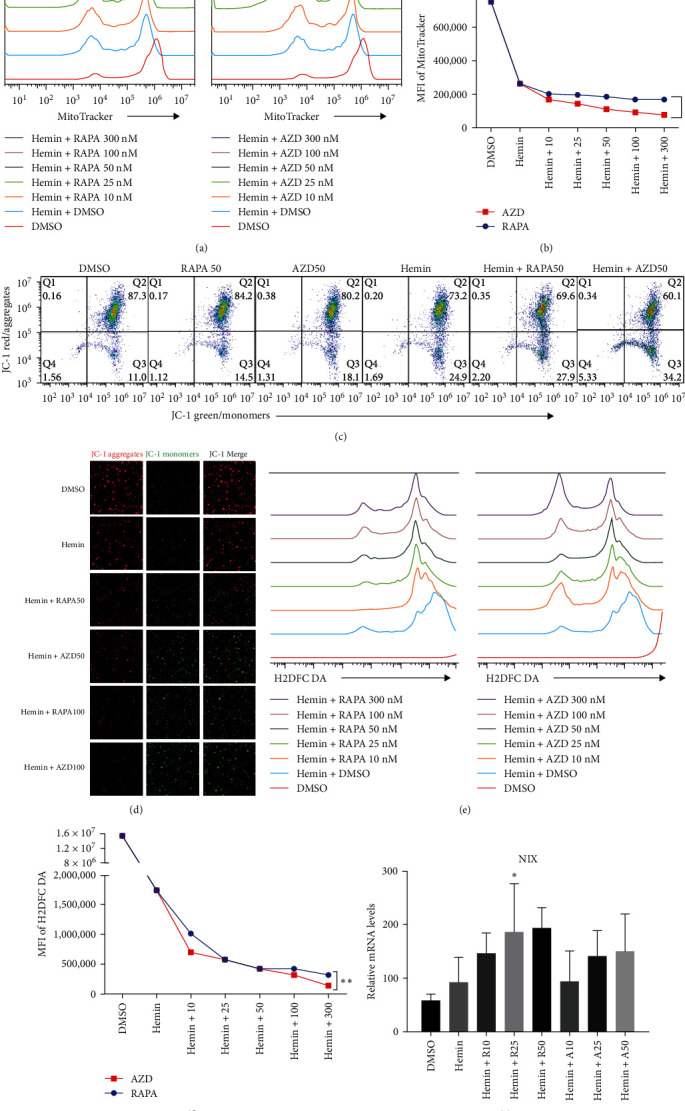
AZD8055 promotes more mitochondrial clearance than rapamycin. (A) The mitochondrial content of K562 cells was evaluated using flow cytometry after labeling with MitoTracker and treatment with rapamycin or AZD8055. (B) Mean fluorescence intensity (MFI) of CFDA SE. (C) The mitochondrial membrane potential changes in K562 cells were evaluated using flow cytometry after treatment with rapamycin and AZD8055 with or without hemin. (D) Confocal laser scanning microscopy images showing the loss of mitochondrial membrane potential demonstrated by the change in JC-1 fluorescence from red (JC-1 aggregates) to green (JC-1 monomers). (E) Intracellular ROS levels as measured via H2DCF-DA staining. (F) Mean fluorescence intensity (MFI) of H2DCF-DA, as analyzed using flow cytometry. (G) *NIX* mRNA expression in K562 cells treated with rapamycin and AZD8055 analyzed using RT-qPCR. Data are the mean ± SD of technical triplicates from one of several independent experiments.  ^*∗*^*P* < 0.05,  ^*∗∗*^*P* < 0.01 vs. K562 cells treated with hemin.

The mitochondria are eliminated during erythrocyte terminal differentiation. In mice, delayed mitochondrial clearance induced oxidative stress and enhanced hemolytic destruction of erythrocytes [12]. To assess the variations in mitochondrial quantity during erythroid differentiation, we labeled K562 cell mitochondria with MitoTracker Green and performed semiquantitative analysis using flow cytometry (Figure 4A, B). The profile of mitochondrial quantity demonstrated that both the rapamycin and AZD8055 groups showed significant decreasing trends with increasing concentration, particularly in the AZD8055-treated group, because the MFI of mitochondria was significantly lower than that in the rapamycin-treated group at the same concentration.

The MMP evaluated with the JC-1 probe was determined using the ratio of red to green fluorescence. Hemin induces mitochondria depolarization [13]. Hemin produced a decrease in MMP compared with the control after rapamycin and AZD8055 treatment (Figure 4C), whereas pairwise comparisons at the same concentration showed that AZD8055 treatment had a more significant effect than rapamycin treatment. K562 cells treated in the same conditions mentioned above were analyzed using confocal microscopy (Figure 4D) to corroborate this observation.

The mitochondria are the main source of ROS [14]; hence, a decrease in ROS is also correlated with a reduction in mitochondria. Rapamycin can scavenge mitochondrial ROS in mice to recover erythrocyte numbers and hemoglobin levels [12]. As the concentration of the treatments increased, the ROS levels in hemin-K562 cells were decreased; however, the effect of AZD8055 treatment was more marked than that of rapamycin treatment (Figure 4E, F).

*NIX* expression increases during erythrocyte maturation and is required for mitochondria degradation upon hemin-induced autophagy. Hemin increased the expression levels of *NIX* compared with the control group, and rapamycin and AZD8055 further increased *NIX* expression (Figure 4G).

### 3.5. Rapamycin and AZD8055 Increase Erythroid Cell Maturation in β-Thalassemia Mice

**Figure 5:**
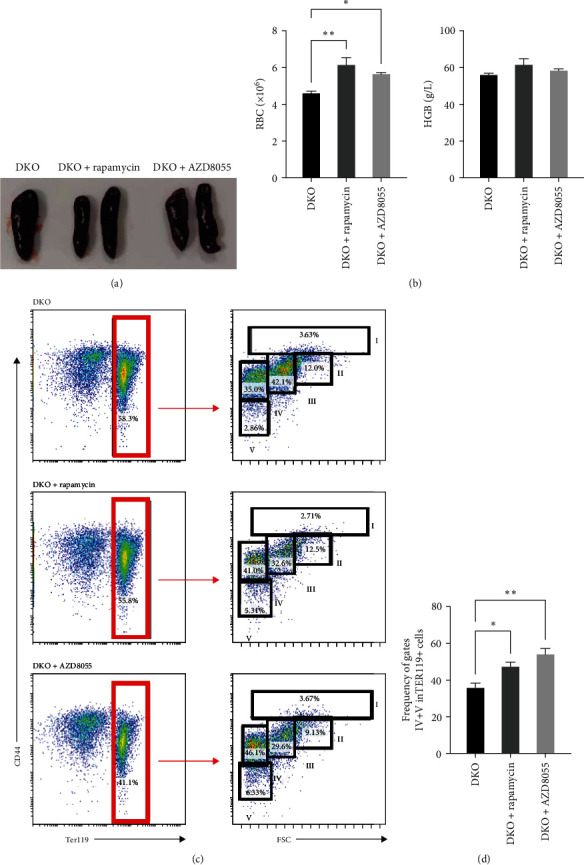
Rapamycin and AZD8055 promote erythroid maturation in β-thalassemia mice. (A) Comparison of spleen morphology in Hbb-bs&Hbb-bt mice treated with vehicle, rapamycin, or AZD8055. (B) Red blood cell number and Hb in Hbb-bs&Hbb-bt mice treated with vehicle, rapamycin, or AZD8055. (C) Flow cytometry analysis of five distinct erythroid populations according to their TER119 and CD44 surface expression and FSC properties. Cells from gate I are proerythroblasts. Cells from region II are basophilic erythroblasts. Cells from region III are polychromatic erythroblasts. Initial sorting of region IV are mixed populations of orthochromatic erythroblasts and immature reticulocytes. Cells from region V are predominantly mature red cells. (D) Ratio of mature cells (gates IV and V combined) relative to the total TER1191 erythroid population. Data are mean ± SD. Data are the mean ± SD of technical triplicates from one of several independent experiments.  ^*∗*^*P* < 0.05,  ^*∗∗*^*P* < 0.01.

Next, we would like to know if rapamycin and AZD8055 promotes erythroid differentiation in β-thalassemia. Rapamycin and AZD8055 treatment resulted in markedly shrinking the spleen and improving red cell numbers compared with baseline in Hbb-bs&Hbb-bt DKO mice (Figure 5A, B). In vivo treatment with rapamycin and AZD8055 increased hemoglobin concentration, but the difference was not statistically significant (Figure 5B). Bone marrow erythroid cells were sorted on the basis of Ter-119 and CD44 expression levels and cell size to distinguish different erythroblast populations [15]. Rapamycin and AZD8055 treatment increased the ratio of mature cells and tipped the balanced production of β-thalassemia erythroid cell towards terminal maturation (Figure 5C, D). These data indicate that rapamycin and AZD8055 enhances β-thalassemia erythropoiesis and promotes erythroid maturation.

### 3.6. Greater Induction of Autophagy by AZD8055 Than That With Rapamycin

**Figure 6:**
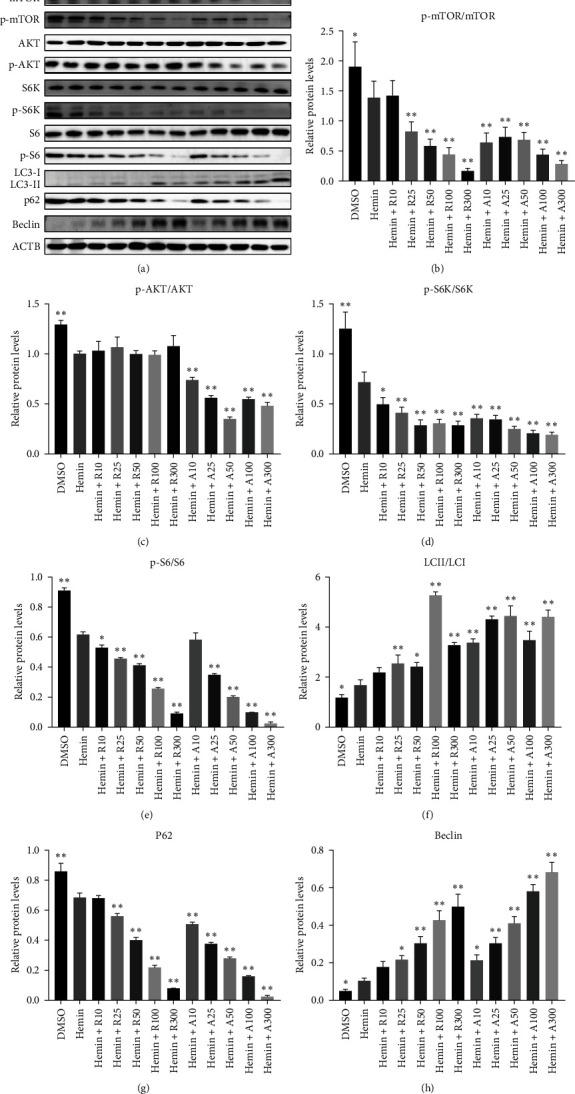
mTOR pathway suppression and autophagy induction were more effective with AZD8055 than that of rapamycin. (A) Western blot analysis for mTOR signaling proteins (p-mTOR (Ser2448), mTOR, p-AKT (Ser473), Akt, p-S6K, S6K p-S6, and S6) and autophagy-related proteins (LC3, p62, and Beclin). (B–H) Protein expression was quantified by densitometry and normalized to β-actin expression. Data are the mean ± SD of technical triplicates from one of several independent experiments.  ^*∗*^*P* < 0.05,  ^*∗∗*^*P* < 0.01 K562 cells treated with. RAPA, rapamycin. A, AZD8055.

mTORC1 primarily contains mTOR with phosphorylated Ser2448, whereas mTORC2 contains mTOR with phosphorylated Ser2481. The mTORC1 complex directly phosphorylates p70S6K on T389, phosphorylating the ribosomal protein S6 on S240/244. mTORC2 activates AKT directly by phosphorylating serine 473. To confirm that mTOR signaling is inhibited upon treatment with rapamycin and AZD8055, the levels of mTOR pathway-related proteins, mTOR, p-mTOR (Ser2448), Akt, p-Akt (Ser473), S6K, p-S6K, S6, and p-S6, were measured using western blotting (Figure 6).

mTOR pathway suppression was more effective with AZD8055 than that rapamycin, as demonstrated by more complete downregulation of phosphorylated pathway members (p-mTOR (Ser2448), p-Akt (Ser473), p-S6K, and p-S6) in a dose- and time-dependent manner. Moreover, only AZD8055 inhibited the phosphorylation of Akt at Ser473, a downstream target of mTORC2. These findings are consistent with the feedback activation of mTOR signaling in response to mTORC1 inhibition, as previously reported [8, 16, 17]. These results indicate that AZD8055 and rapamycin can suppress the mTOR pathway; however, rapamycin inhibits only mTORC1, whereas AZD8055 inhibits both mTORC1 and mTORC2.

Both rapamycin and AZD8055 can induce autophagy [18]; hence, we evaluated whether they would also affect this process during the erythroid differentiation of hemin-induced K562 cells. LC3I-II conversion, upregulation of the autophagy-related protein Beclin-1, and downregulation of p62 were used to verify autophagy induction. Western blotting demonstrated that LC3II and Beclin-1 increased, whereas p62 decreased dose-dependently following AZD8055 and rapamycin treatment (Figure 6). Autophagy was significantly increased with AZD8055 compared to that with rapamycin at the same concentration. These results indicate that both treatments can enhance autophagy; however, the effect is more effective by AZD8055.

## 4. Discussion

Previous studies demonstrated the different roles of mTORC1 and mTORC2 in cardiomyocyte differentiation from mES cells in vitro [19], NK cell development [20], and γδ T cell differentiation [7]. We also investigated the effect of mTORC1 on erythroid differentiation, but the role of mTORC2 remains unclear. We investigated the selective mTORC1 and dual mTORC1/mTORC2 inhibitors, rapamycin and AZD8055, respectively. AZD8055 was more potent than rapamycin in inhibiting cell proliferation and clearing mitochondria. We further investigated the underlying molecular mechanism and found that AZD8055 significantly enhanced autophagy compared with rapamycin. Therefore, we identified the functions of mTORC1 and mTORC2 in erythroid differentiation.

This study compared AZD8055 and rapamycin in hemin-induced K562 cells, observing greater growth inhibition with AZD8055. The inhibitory mechanism for both AZD8055 and rapamycin included cell cycle arrest, consistent with the effect reported in chronic lymphocytic leukemia cells [21]. AZD8055 decreased cell proliferation and cell cycle progression and reduced the clonogenic growth of leukemic progenitors [16]. These results are consistent with the report that AZD8055 induces G0/G1 cell cycle arrest, reduces cyclin D1, and increases *p27* expression in colon cancer cells [22], adult T-cell leukemia cell lines [23], and T cells [24]. Dual targeting of mTORC2/mTORC1 in leukemic cells results in potent suppressive effects on primitive leukemic progenitors from chronic myelogenous leukemia patients [25]. The target of rapamycin (TOR) protein kinase plays a pivotal role in metabolism and gene expression, which enables cell proliferation, growth, and development [26]. AZD8055 also induces a dose-dependent growth inhibition and/or regression in lung cancer cells, head and neck squamous cell carcinoma [27], cervical cancer [28], and colorectal cancer [29], which is associated with the dose-dependent pharmacodynamic effect on pS6 and pAKT [30].

AZD8055- and rapamycin-induced expression of erythroid markers (α-, ε-, and γ-globin and CD235a). ε-Globin mRNA showed the greatest increase with hemin induction, followed by ζ, γ, and α in K562 cells [31]. Rapamycin is a powerful inducer of erythroid differentiation and γ-globin mRNA accumulation in human leukemia K562 cells [32]. Low doses of sirolimus can modify hematopoiesis and induce increased expression of γ-globin genes in a subset of patients with β-thalassemia [33]. Raptor, an important companion of mTOR in the mTORC1 complex, is reported to be involved in erythroid differentiation of K562 cells and participate to control of γ-globin gene expression in erythroid precursor cells [34]. Although AZD8055 promoted globin expression, its effect was not significantly different from rapamycin.

The mitochondria are eliminated during the late stage of erythrocyte maturation. Delayed mitochondrial clearance in mice can induce oxidative stress and enhance the hemolytic destruction of erythrocytes [12]. AZD8055 and rapamycin, especially AZD8055, significantly reduced mitochondrial content. Modifications in mitochondrial shape and/or number in leukemic cells participate in chemoresistance and may be related to their proliferative potential [35]. In Down syndrome, AZD8055 can restore the clearance of damaged mitochondria [36]. Mitochondria membrane depolarization (MMP), an important signal to the clearance of this organelle, is characteristic of mitochondrial dysfunction [37] and can be induced by hemin in K562 cells similar to that with mitophagy [13, 38].

Furthermore, we presented evidence that hemin produces significantly reduced MMP after rapamycin and AZD8055 treatment, especially with AZD8055 treatment. Alterations in ROS production and MMP are characteristics of mitochondrial dysfunction. The potent antioxidant agent reduced intracellular ROS levels, thus restoring differentiation to mature erythroblasts [39]. Our results showed that intracellular ROS levels were significantly decreased after rapamycin and AZD8055 treatment, especially the latter. ROS levels are elevated in leukemic cells compared with normal cells, leading to differing biological outcomes [40]. Rapamycin scavenges mitochondrial ROS to alleviate hemolytic destruction and anemia in mice [12]. During erythroid differentiation of hemin-induced K562 cells, AZD8055 may remove mitochondrial ROS to improve erythrocyte maturation. *NIX*, upregulated during reticulocyte maturation, triggers mitochondrial depolarization [6, 41, 42]. We found that *NIX* expression was increased following AZD8055 and rapamycin treatment. *NIX*, which is necessary for the engulfment of mitochondria within the autophagosomes upon hemin-induced autophagy [41–43], is upregulated by AZD8055 and rapamycin. Thus, AZD8055 may promote mitochondrial clearance and reduce intracellular ROS production through *NIX* upregulation.

An animal model would allow for a better understanding of the in vivo implications of rapamycin and AZD8055. β-Thalassemia is a disease characterized by anemia and ineffective erythropoiesis. In β-thalassemia model mouse, excessive compensatory proliferation of erythroid precursors that fail to mature leads to ineffective erythropoiesis [44]. We found both drugs improved anemia and erythroid cell maturation and diminished ineffective erythropoiesis. In our previous research, in vivo rapamycin remarkably improves erythroid cell maturation and anemia in a model of β-thalassemia [45]. Recently, sirolimus has been considered in clinical studies to find novel protocols for the therapy of β-thalassemia [46]. The improvement of β-thalassemic ineffective erythropoiesis is associated with diminished mTOR activation and improved autophagy [47]. AZD8055 is an orally bioavailable, potent, and selective TOR kinase inhibitor that binds to the ATP binding cleft of TOR kinase and inhibits both TORC1 and TORC2. AZD8055 as a treatment modality for ineffective erythropoiesis in preclinical model systems is to our knowledge novel and in our opinion of interest, providing a strong scientific basis for further clinical trials.

During erythropoiesis, autophagy plays an important role in the clearance of unnecessary organelles, such as mitochondria (mitophagy), thereby allowing the correct formation of mature erythrocytes. Rapamycin plays a dual role in neuroprotective effects via the stimulation of autophagy, which leads to damaged mitochondria removal and enhancement of mitochondria fission to facilitate their elimination by mitophagy. After rapamycin and AZD8055 treatment, autophagy genes were enhanced, and mitochondrial clearance was increased, demonstrating the association between autophagy and mitochondrial removal. Hemin promotes autophagy in K562 cells [13]. Consistent with this, we confirmed that hemin produced an increased expression in autophagy-associated proteins, such as LC3II, Beclin-1, and a decreased expression level of p62, which was further increased after rapamycin and AZD8055 treatment. AZD8055 and rapamycin-induced autophagy has also been reported in K562 and other cell models [16, 18].

The mTOR signaling pathway can induce autophagy by modulating the formation of erythrocytes downstream [6]. Hemin induces the activation of the autophagic pathway via mTOR signaling. Although rapamycin is an allosteric inhibitor of mTORC1 and only induces a sustained inhibition of p70S6K and S6K phosphorylation, it is uncoupled by the negative feedback loop mediated by mTORC1 on mTORC2, enabling AKT phosphorylation. Our findings are consistent with the feedback activation of mTOR signaling in response to mTORC1 inhibition, as previously reported [8, 16, 17]. AZD8055 has a more marked effect on mTORC1 than rapamycin and inhibits the mTORC2 pathway. However, further studies are required to identify the specific role of mTORC2 in erythroid differentiation.

## 5. Conclusions

We demonstrated that the dual mTORC1/mTORC2 inhibition by AZD8055 inhibited the proliferation and promoted mitochondrial clearance during erythroid differentiation of hemin-induced K562 cells more effectively than the selective mTORC1 inhibitor rapamycin. The role of mTORC1 and mTORC2 in erythropoiesis suggests that AZD8055 may provide a better therapeutic strategy than rapamycin and analogs in the differentiation-inducing treatment of chronic myelogenous leukemia.

## Data Availability

The data that supports the findings of this study are available within the main text and supporting information of this article.

## Supporting Information

Supporting Information Table S1: The primer sequences for the real-time PCR are listed. The primer sequences for the real-time PCR are listed in Table 1.
