# Supplementary material for: AZD8055 Is More Effective Than Rapamycin in Inhibiting Proliferation and Promoting Mitochondrial Clearance in Erythroid Differentiation
Source: Anal Cell Pathol (Amst). 2024 Oct 8;2024:2639464. doi: 10.1155/2024/2639464 (PMC11479778; doi:10.1155/2024/2639464)
Supplement: Supporting Information — Table S1: The primer sequences for the real-time PCR are listed. The primer sequences for the real-time PCR are listed in Table 1. [file 2639464.f1.docx]

**Supplementary Table 1: Real-time polymerase chain reaction primers**

|  | **Primer sequence** | |
| --- | --- | --- |
| *p27* | Forward primer | 5’-CGGCTCATGGGCGACTATC-3’ |
|  | Reverse primer | 5’-TGTCTTGGAGGAGGATCGTCC-3’ |
| *a-globin* | Forward primer | 5’-GGTCAACTTCAAGCTCCTAAGC-3’ |
|  | Reverse primer | 5’-GCTCACAGAAGCCAGGAACTTG-3’ |
| *ε-globin* | Forward primer | 5’-CAGCTGCAATCACTAGCAAGC-3’ |
|  | Reverse primer | 5’-AGACGACAGGTTTCCAAAGC-3’ |
| *γ-globin* | Forward primer | 5’-AACCCCAAAGTCAAGGCACA-3’ |
|  | Reverse primer | 5’-CAAAACGGTCACCAGCACAT-3’ |
| *NIX* | Forward primer | 5’-CTCACTGTGACAGCCCTTCG-3’ |
|  | Reverse primer | 5’-ACCCAGTCCGCACTTTTCTT-3’ |
| *ATCB* | Forward primer | 5’-TCGTGATGGACTCCGGTGAC-3’ |
|  | Reverse primer | 5’-TCGTGGATGCCACAGGACTC-3’ |
